# Bridging the Gap in Carbohydrate Counting With a Mobile App: Needs Assessment Survey

**DOI:** 10.2196/63278

**Published:** 2025-03-28

**Authors:** Asmaa Housni, Alexandra Katz, Lucien Junior Bergeron, Alain Simard, Ashley Finkel, Amélie Roy-Fleming, Meranda Nakhla, Anne-Sophie Brazeau

**Affiliations:** 1 School of Human Nutrition McGill University Montreal, QC Canada; 2 Faculté de médecine Université de Montréal Montreal, QC Canada; 3 Faculté de Médecine et des Sciences de la Santé Université de Sherbrooke Sherbrooke, QC Canada; 4 Petit Cactus Inc Sherbrooke, QC Canada; 5 Research Institute of the McGill University Health Center Division of Endocrinology Montreal Children's Hospital Montreal, QC Canada

**Keywords:** type 1 diabetes, carbohydrate counting, mobile apps, photo recognition, diabetes management, mHealth, mobile health, user-centered design, Canadian survey

## Abstract

**Background:**

Carbohydrate counting (CC) can be burdensome and difficulty with adherence has been reported. Automated CC through mobile apps offers innovative solutions to ease this burden.

**Objective:**

This cross-sectional web-based survey aims to identify (1) perceived barriers to CC by Canadians living with type 1 diabetes (T1D) and (2) app features that would help reduce these barriers. The secondary objective aims to compare apps used by participants with the suggested app features.

**Methods:**

People with T1D aged 14 years and older, living in Canada, were recruited through the BETTER Canadian registry, diabetes organizations, and social media. Participants completed a 39-question web-based survey (closed- and open-ended) to identify barriers in CC, preferred CC app features, and current app use. Respondents rated barriers and app features using a 5-point Likert scale. The features were cross-referenced in each app reported being used by participants. Descriptive statistics summarized barriers and app feature preferences, and statistical analyses identified differences by age, app use, and insulin modality. Mean scores (out of 5) were compared using 2-tailed *t* tests or nonparametric tests. Open-ended questions were analyzed using inductive thematic analysis.

**Results:**

Participants (N=196; woman: n=145, 74%; mean age 40 [SD 17] years; mean diabetes duration 22 (14) years; relied on CC to determine insulin doses at mealtimes: n=178, 90.8%) reported barriers related to carbohydrate identification, nutrient interaction, and insulin dose calculation, as well as psychosocial factors. Preferred app features included nutrient analysis (165/196, 84.2%), personalization (151/196, 77.1%), insulin bolus calculation (145/196, 74%), and health care professional support (135/196, 68.8%). Among the 16 apps used by participants, most (12/16, 75%) supported nutrient analysis but only one offered bolus calculations or health care professional support, and none offered personalization. Users on injections reported greater barriers to blood glucose monitoring for insulin adjustments compared to exclusive pump users (mean score of 3.87, SD 1.22 vs mean 3.30, SD 1.28; *P*=.001). They also expressed higher needs for meal logs in an electronic food journal (mean 4.06, SD 1.18 vs mean 3.69, SD 1.17; *P*=.01), bolus dose suggestions (mean 4.37, SD 0.98 vs mean 3.84, SD 1.26; *P*=.001), and app personalization (mean 4.47, SD 0.86 vs mean 3.93, SD 1.21; *P*<.001). No significant differences were observed based on age or app use. The thematic analysis revealed participants’ perceptions of suggested barriers and features, as well as new barriers such as calculation errors from unreliable food data and nutrition labels, fear of eating disorders, limited app reliability, and insufficient health care support, with suggestions for technology-based solutions.

**Conclusions:**

CC mobile apps currently used do not meet the needs of people with T1D. A novel CC app with app features such as photo recognition, reliable nutrient values, and personalized bolus calculations could reduce the CC burden.

## Introduction

People living with type 1 diabetes (T1D; approximately 300,000 in Canada) need to be administered subcutaneous insulin to maintain stable blood glucose (BG) levels and prevent long-term complications [1,2]. Instead of a fixed bolus insulin regimen tied to predetermined meal plans, carbohydrate counting (CC) offers dietary flexibility by empowering individuals to tailor their mealtime insulin based on the carbohydrate content of their meals [3]. However, adherence to CC can be burdensome and difficult [4]. CC requires frequent BG monitoring, keeping food records, reading food nutrition labels, and weighing food portions. Additionally, calculations are time-consuming and prone to errors as they involve multiple factors such as the insulin sensitivity factor, insulin-to-carbohydrate ratio, and BG target [5,6]. Accurate CC is important because greater precision in carbohydrate counting improves insulin dosing accuracy and glycemic management, leading to lower glycated hemoglobin levels [7]. Further, greater differences in carbohydrate estimates are associated with higher glycemic variability [8]. This is significant considering that frequent or large glucose fluctuations may independently contribute to diabetes-related complications [9].

Automated CC through health technology introduces novel care solutions that can simplify CC for individuals living with T1D. The efficacy of smartphone or tablet apps in improving self-management among adults with diabetes is well-established [10-12]. The use of automated carbohydrate estimation compared to conventional methods (ie, manual calculations) led to improved accuracy in CC, reduced time spent in hyperglycemia, and improved BG variability [13,14]. However, the use of such apps is limited beyond research settings [15,16]. According to a survey, young participants lack awareness and have skepticism about the effectiveness of diabetes management apps, which have been shown to be reasons for not using them [16].

This highlights the necessity of involving people with T1D in the design process to create a more effective and user-centered app design that meets their needs. This is particularly important for adolescents, considering the unique psychological and social challenges they face [17]. Consequently, they may struggle to maintain motivation for self-care behaviors such as CC, as competing priorities often take precedence over the numerous demands of T1D management [18]. This challenge is compounded by the increased glycemic levels disproportionately observed from adolescence through young adulthood (approximately aged 14-24 years) [19]. Recognizing the vulnerability of this developmental stage, during which adolescents and young adults must develop new skills and competencies, it is essential to include adolescents in the development of new resources to gain insights into their specific needs [20].

To understand how a CC app can facilitate CC at mealtimes for people with T1D, the primary objectives of our study are to identify (1) the perceived barriers to CC and (2) the app features that would help reduce these barriers. The secondary objective is to compare apps being used by participants with the suggested app features to identify gaps for a novel CC app that aligns with user preferences and needs. We hypothesized that the needs of people with T1D regarding CC are not met with traditional manual tools or with the currently used apps.

## Methods

### Ethical Considerations

We received ethics approval for this study from the McGill Research Ethics Board (22-08-054-02). Participants were informed through the web-based platform (LimeSurvey GmbH) before accessing the survey, about its estimated length, the type of data collected, where the data would be stored, the duration of storage, the investigator’s identity, and the purpose of the study. Informed consent was obtained before participation. All data collected were deidentified to ensure privacy and confidentiality and were hosted on secure McGill servers. No compensation was offered for completing the survey.

### Participants

A national cross-sectional web-based survey was launched across Canada. Recruitment took place from November 2022 to November 2023 and used a nonprobability convenience sample. We sent an announcement through the newsletter to participants of the BETTER Canadian registry of people living with T1D (more than 3800 participants) [21] and through Canadian diabetes organizations and social media. People with T1D diagnosis, aged 14 years and older, and living in Canada, were eligible to complete a 30-closed and 9-open-ended question survey. Adolescent and adult participants provided digital consent before accessing the open voluntary survey. Parents who completed the questionnaire on behalf of their child with T1D were excluded as the aim was to gather insights and perceptions directly from individuals living with T1D.

### Questionnaire and Data Collection

All data were self-reported. The survey was developed and adapted by researchers of T1D, certified diabetes educators, and health care professionals on the team, and subsequently tested by 3 patient-partners (ie, individuals with lived experience of type 1 diabetes who collaborate in research, to ensure a clear understanding of the questions). Questions related to barriers in CC were based on a literature review and proposed app features were based on the scientific literature as well as a manual search or testing of iOS and Android diabetes management apps. A list of identified barriers and proposed app features was compiled, and consensus was reached among researchers (AH and ASB) for the final items included in the survey. The survey was created, in both English and French, on LimeSurvey hosted on McGill secure servers (LimeSurvey GmbH). The final questionnaire contained 13 demographic and diabetes management–related nonrandomized questions, 19 CC needs assessment questions and app use, and 7 questions on patient-provider communications, distributed over 6 pages or screens (Multimedia Appendix 1). Adaptive questioning was used to reduce the number and complexity of the questions. Respondents were able to review and change their answers before submission. The survey design ensured single-entry responses by disabling token-based response persistence and prohibiting multiple or updated responses with the same token. Only the completed questionnaires were considered for analysis. Respondents rated suggested barriers and app features on a 5-point Likert scale. The percentage of agreement was calculated based on the frequency of ratings that were either a 4=agree or a 5=strongly agree. Suggested app features were cross-referenced with the features of apps reported by participants to identify gaps and inform the development of a novel CC app aligned with user preferences and needs.

### Statistical Analyses

All analyses were performed using IBM SPSS Statistics (version 27; IBM Corp). Variables are reported as frequencies, median with IQR, or mean (SD) was computed where indicated for scale variables. In addition to descriptive statistics for presenting barriers and app feature preferences, statistical analyses were conducted to identify any significant differences among respondents based on age (youths aged 14-24 years vs adults older than 24 years), consistent app use (every day or most of the days vs some of the days or rarely), and insulin administration modality (on injections or sometimes on an insulin pump vs on an insulin pump exclusively). A mean score out of 5 was calculated for each barrier and app feature and treated as a continuous variable to assess mean differences using either a 2-tailed *t* test or a nonparametric test, as appropriate. Open-ended questions were analyzed by 2 independent researchers (AH and AK), both with experience in diabetes care as clinicians (AH is a registered dietitian and AK is an MD student) and as student researchers. Their familiarity with the challenges faced by individuals with T1D and in conducting CC informed their interpretation of the open-ended responses. MAXQDA software (version 24; VERBI GmbH) was used to assist with the inductive thematic analysis and coding process. Individually, each researcher created a codebook, and then each code was discussed until agreement (100%) was achieved.

## Results

### Overview

A final sample of 196 eligible respondents provided consent and completed all the questions of the survey. The majority identified as women (n=145, 74%) and Caucasian (n=184, 93.9%). The median (IQR) age was 38 (24-54) years with just over a quarter of participants being youths aged 14-24 years (n=52, 26.5%). Over half of the respondents (n=110, 56.1%) use an insulin pump to administer insulin and almost all participants (n=191, 97.4%) use continuous glucose monitoring (CGM) systems (Table 1). Most respondents (n=178, 90.8%) use CC to determine mealtime bolus insulin and more than half (n=133, 67.9%) find it difficult to manage BG levels around mealtime with CC. Although the majority (n=147, 7%) believe apps could ease the CC burden, only 57 (29.1%) use apps to help with CC, and less than half of them (27/57, 47.4%) are satisfied with the apps used. A total of 16 unique apps were reported as being used by the respondents (Table 2). The most frequently used app was “MyFitnessPal,” reported by 17 of the 57 participants. This was followed by “Carbs & Cals,” “BolusCalc,” and “CalorieKing,” each used by 3 respondents.

**Table 1:** Participant (n=196) characteristics.

Characteristics					Values
**Demographics**					
		**Age (years), median (IQR)**		38 (24-54)	
			Youths (14-24), n (%)	52 (26.5)	
			Adults (25 and older), n (%)	144 (73.5)	
		Genderª (women), n (%)		145 (74)	
		Born in Canada, n (%)		176 (89.8)	
**Ethnicity, n (%)**					
		Asian		4 (2)	
		Arab		3 (1.5)	
		Black (African, African American, and Caribbean)		1 (0.5)	
		Latin American		2 (1)	
		White or Caucasian		184 (83.9)	
		Other (Iberian and mixed)		2 (1)	
Currently studying, n (%)					47 (24)
**Highest level of education acquired, n (%)**					
		High school level		40 (20.4)	
		Cégep, vocational, or community college		58 (29.6)	
		University levelᵇ		98 (50)	
**Medical history and diabetes management, median (IQR)**					
	Diabetes durationᶜ (years)			19 (12-32)	
**Had at least 1 diabetes-related consultation in the last year with health care professionals, n (%)**					
	Medical specialist (endocrinologist, pediatrician, and internist)			185 (94.4)	
	Family doctor or general practitioner			97 (49.5)	
	Registered dietitian or nutritionist			69 (35.3)	
	Registered nurse			64 (32.6)	
	Other (psychologist, dentist, or ophthalmologist)			50 (25.5)	
Use continuous glucose monitoring systems in the last 12 months, n (%)					191 (97.4)
Use insulin pens or syringes exclusively, n (%)					86 (43.9)
Use an insulin pump, n (%)					110 (56.1)

^a^n=1 identifies as genderfluid, n=49 as men, n=1 missing..

^b^University certificate, bachelor’s degree, master’s, PhD, or MD.

^c^n=2 missing.

**Table 2:** Suggested app features were ranked by rate of agreement (4 or 5 on the Likert scale), then cross-referenced in each app (n=16) reported to be used by participants. The checkmarks indicate the features provided by the app.

Suggested app features	Values, n (%)	Carb Manager	Foodvisor	Lose it	MyFitnessPal	Cronometer	FatSecret	Fitwell	SNAQ^a^	Figwee	Carbs & Cals	Glycemeal	Keenoa	Bolus Calc	Calorie King	Glucicheck	MySugar
Viewing the food nutrition label	167 (85)	✓	✓	✓	✓	✓			✓						✓		
Meal composition quantification	165 (84)	✓	✓	✓	✓	✓	✓	✓	✓	✓	✓	✓	✓				
Personalization of the app	151 (77)																
Suggestion of insulin bolus doses	145 (74)													✓			
Unrestricted Support from health care professionals	135 (69)		✓														
Meal logs into a food journal	129 (66)	✓	✓	✓	✓	✓	✓	✓	✓	✓	✓	✓	✓				
Community building with app users	93 (47)	✓	✓	✓	✓		✓	✓									
Gamification	79 (40)	✓		✓	✓												

^a^SNAQ: Simplified Nutritional Appetite Questionnaire.

**Figure 1:**
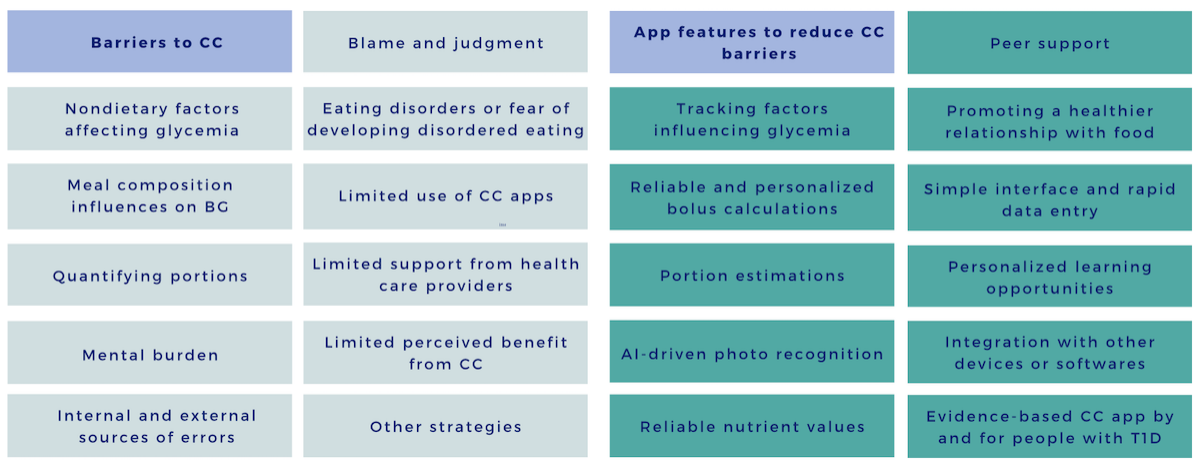
Emerging themes of reported barriers to carbohydrate counting (n=11) and app features (n=11) perceived to reduce these barriers, from the qualitative analysis of the open-ended questions. AI: artificial intelligence; BG: blood glucose; CC: carbohydrate counting; T1D: type 1 diabetes.

The open-ended survey responses were analyzed using inductive qualitative thematic analysis, identifying 22 themes categorized as barriers to CC and app features to address these barriers (Figure 1). Although some themes were survey options, participants also introduced new barriers and features. Emergent themes included calculation errors due to unreliable food data and nutrition labels, limited app reliability, unmet diverse user needs, and fear of eating disorders associated with food hyperfixation. Concealing calorie information through technology was suggested as a way to reduce this hyperfixation. Participants highlighted minimal perceived benefits of CC on BG trends and limited health care professionals’ support due to time constraints, lack of nutrition knowledge, and narrow clinical focus. Alternative strategies were brought forward to avoid CC, such as avoiding carbohydrates, using CGM to correct BG, eating the same thing, or using trial and error.

### Barriers to CC

**Figure 2:**
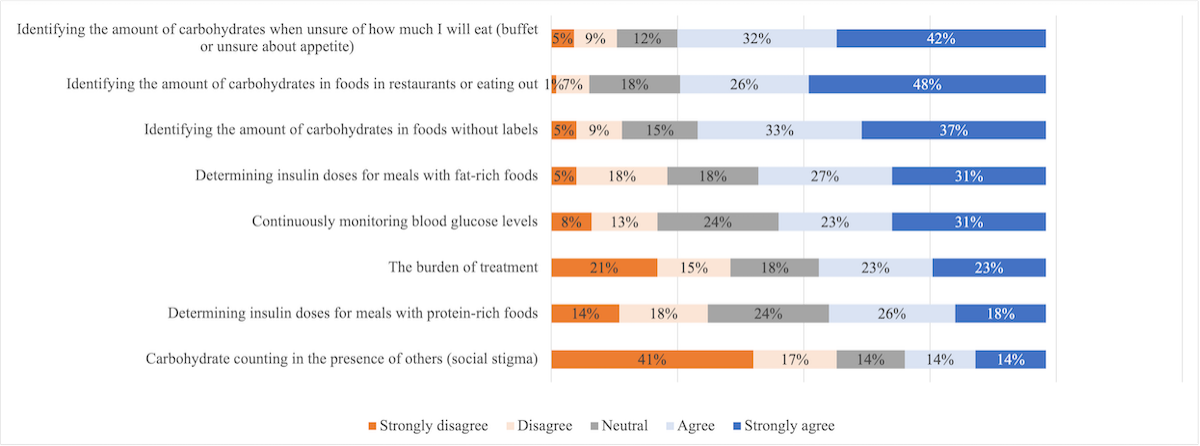
Stacked bar chart of participants’ proportion of agreement with suggested barriers to carbohydrate counting.

In terms of the barriers to CC that were suggested, identifying the number of carbohydrates to be consumed was the most agreed upon barrier whether it was in foods without labels (n=138, 70.4%; ie, rated scores of 4 or 5), in restaurants or when eating out (n=145, 74%), or when unsure about appetite (n=146, 74.4%; Figure 2). This was also reflected in participants’ answers to the open-ended questions where quantifying portions was reported as a barrier for CC when eating homemade food or unknown ingredients, but also for food with labels where the serving size on nutrition labels was unclear or difficult to estimate. Conversely, counting CC in the presence of others or social stigma achieved higher rates of disagreement (n=56, 57.6%; ie, rated scores of 1 or 2). However, qualitative data analysis revealed a distinct perspective. Specifically, stigma manifested as feelings of blame and judgment. Participants expressed a fear of alienation, as well as apprehension about receiving judgment when eating sugary foods. It also included perceptions of stigma stemming from preoccupation with others' opinions, including health care professionals, and the fear of “ruining a good day” (Figure 1).

### App Features to Reduce CC Burden

Suggested app features with the most agreements were the option to view the food nutrition label (n=167, 85.2%) and the quantification of meal composition (n=165, 84.2%). Participants expanded upon this in the open-ended responses by suggesting photo recognition, visual cues, comparing pictures to estimate quantities, or shifting toward the selection of small-medium-large portions rather than exact grams to reduce the burden. Beyond estimating quantity, emphasis was also placed on reliable nutrient value by suggesting the inclusion of the source of nutrient information and a breakdown of the carbohydrate estimations (Multimedia Appendix 2). On the other hand, gamification features such as earning points or trophies achieved the most disagreement among respondents (n=75, 38.3%; Table 3). To simplify meal logging and keeping a food journal, the majority of respondents agreed on adding a “Favorites” section (n=168, 85.7%), allowing users to save frequently consumed food items for convenient access and photo recognition features (n=156, 79.2%). Additionally, a significant majority (n=166, 84.7%) agreed on including options to log factors influencing glycemia such as alcohol intake, physical activity, and medications (Table 3). In the open-ended responses, participants also noted stress, sick days, menstrual cycle, sleep, time, and site of injections, as well as other medical factors (menopause and gastroparesis; Figure 2). Factors that would increase users’ trust in CC apps include transparency about safety mechanisms to prevent accidental overdosing of insulin or hypoglycemia (n=147, 75%) and endorsements based on validated clinical trial results (n=146, 74.5%; Table 2). Additionally, open-ended answers highlighted participants’ emphasis on users’ feedback, consistency in results obtained from the app, and improved glycemic management outcomes (Figure 1).

**Table 3:** Agreement rates for suggested app features and factors reducing CC burden.

Suggested app feature		Rate of agreement, n (%)				
		1=strongly disagree	2=disagree	3=neutral	4=agree	5=strongly agree
**Features that would be beneficial to include in a carbohydrate counting app**						
	Gamification (earning points or trophies)	50 (25.5)	25 (12.8)	42 (21.4)	40 (20.4)	39 (19.9)
	In-app community building	17 (8.7)	31 (15.8)	55 (28.1)	40 (20.4)	53 (27)
	Meal logs into a food journal	11 (5.6)	15 (7.7)	41 (20.9)	53 (27)	76 (38.8)
	Support from health care providers	13 (6.6)	13 (6.6)	35 (17.9)	63 (32.1)	72 (36.7)
	Suggestion of insulin bolus doses	11 (5.6)	10 (5.1)	30 (15.3)	46 (23.5)	99 (50.5)
	Personalization of the app	9 (4.6)	7 (3.6)	29 (14.8)	47 (24)	104 (53.1)
	Quantification of meal composition	5 (2.6)	7 (3.6)	19 (9.7)	60 (30.6)	105 (53.6)
	Viewing the food nutrition label	5 (2.6)	4 (2)	20 (10.2)	47 (24)	120 (61.2)
**Features that would facilitate food journaling**						
	Reminders for forgotten logs	27 (13.8)	37 (18.9)	49 (25)	49 (25)	34 (17.3)
	Photo recognition	6 (3.1)	10 (5.1)	24 (12.2)	39 (19.9)	117 (59.7)
	Save meals in a “Favorite” section	5 (2.6)	4 (2)	19 (9.7)	53 (27)	115 (58.7)
	Access recent meal items.	3 (1.5)	4 (2)	29 (14.8)	62 (31.6)	98 (50)
	Combine food items into recipes	4 (2)	5 (2.6)	28 (14.3)	56 (28.6)	103 (52.6)
	Track BG^a^ levels and related meals or insulin	3 (1.5)	10 (5.1)	31 (15.8)	52 (26.5)	100 (51.0)
	Include meal notes and postadjustments	4 (2)	8 (4.1)	37 (18.9)	63 (32.1)	84 (42.9)
	Log factors influencing BG	4 (2)	4 (2)	22 (11.2)	64 (32.7)	102 (52)
**Factors to increase my trust in a carbohydrate counting app**						
	Validated with clinical trials	3 (1.5)	10 (5.1)	37 (18.9)	57 (29.1)	89 (45.4)
	Secure data storage	5 (2.6)	12 (6.1)	49 (25)	41 (20.9)	89 (45.4)
	Information on safety mechanisms	5 (2.6)	12 (6.1)	32 (16.3)	63 (32.1)	84 (42.9)
	Access formulas used for calculations	11 (5.6)	12 (6.1)	44 (22.4)	47 (24)	82 (41.8)
	Qualifications of app developers	9 (4.6)	13 (6.6)	46 (23.5)	47 (24)	81 (41.3)
	Health Canada’s approval	8 (4.1)	11 (5.6)	39 (19.9)	50 (25.5)	88 (44.9)
	Endorsed by the health care team	10 (5.1)	8 (4.1)	43 (21.9)	52 (26.5)	83 (42.3)

^a^BG: blood glucose.

### App Features Comparison With Current Apps

Suggested app features were cross-referenced in each app (n=16) reported to be used by participants. It was found that, while most apps allow meal composition quantification, less than half display nutrition labels. Only 1 app calculated bolus dose, 1 app provided support from health care professionals, and none offered personalization for diabetes characteristics (Table 2).

### Differences in Barriers and App Feature Preferences Among Users

Based on insulin administration modalities, the need to continuously monitor BG levels to adjust insulin ratios was a more pronounced barrier among users on injections (mean score of 3.87, SD 1.22; ie, trending toward agreement) compared to those using insulin pumps exclusively (mean score of 3.30, SD 1.28; ie, trending toward neutral; *P*=.001). Additionally, users on injections expressed a greater need for specific features compared to exclusive pump users. This included the need for meal logs in an electronic food journal (mean score of 4.06, SD 1.18 vs mean 3.69, SD 1.17; *P*=.01), suggestions for bolus doses (mean 4.37, SD 0.98 vs mean 3.84, SD 1.26; *P*=.001), and app personalization (mean 4.47, SD 0.86 vs mean 3.93, SD 1.21; *P*<.001).

There were no significant differences in barriers or app feature preferences based on age or app use.

## Discussion

### Principal Findings

Participants reported carbohydrate identification barriers, nutrient interaction, and insulin dose calculation barriers, as well as psychosocial barriers. App features like photo recognition paired with nutrient values derived from validated databases, personalized bolus calculations, and tracking factors affecting BG levels were perceived to facilitate CC. Existing CC apps, used by people with T1D, fall short of meeting their preferred app features.

### Comparison With Prior Work

Martinez et al [22] found that of the 80 existing apps for people with T1D, none met the criteria of an “ideal” app, suggesting a significant gap between what users want and what is available. App features for general T1D management, such as access to CGM and insulin pump records, personalization through goal setting and tailored recommendations, as well as insulin bolus calculations [22] were also reiterated by participants in this survey. On the other hand, reminders and rewards, that is, gamification features did not obtain major agreement in this survey despite being present in the literature as key features for diabetes management technology [22]. Several novel app features were suggested by participants and are worth noting. In fact, the fear of developing eating disorders was mentioned as a barrier to CC. The hyperfixation on food can lead to a heightened risk of developing disordered eating behaviors among people with T1D and is a well-established risk factor in the literature [23]. As such, concealing calorie information was suggested to promote a healthier relationship with food using technology. This perspective aligns with existing literature that highlights the importance of automated processes, which demand less input from the individual, as a means to alleviate the burden of treatment in T1D. A review demonstrated that the use of automated insulin delivery systems may reduce food management burden, by correcting CC inaccuracy, particularly when combined with adjunctive therapies (eg, GLP-1 receptor agonists and SGLT2 inhibitors) [24,25]. Although automated insulin delivery systems are at the forefront of diabetes research, fully closed-loop systems that do not require CC remain in development and are not yet available for people with T1D [26]. Additionally, factors such as future costs and user preferences underscore the need to provide alternative solutions, particularly accessible, comprehensive, and customizable CC apps. Meal entry through photo recognition could alleviate the cognitive burden on individuals with T1D during mealtimes and potentially facilitate full automation. This would enable users to receive bolus dose suggestions simply by taking a picture of their meal, without needing to process additional information. However, it is important to provide users with the option to access breakdowns of carbohydrate estimations and calculations upon request, as it was found to increase their trust in a CC app. Meal entry through photo recognition would also decrease the time required for CC and could potentially alleviate associated burdens. Participants expanded on the mental strain linked with CC, describing it as time-consuming, challenging to log multiple small snacks and corrections between meals, and restrictive when “not being able to eat right away.” Another key barrier that was introduced was the limited use of CC apps. Beyond the 16 apps reported as being used by respondents, other available apps did not meet the reported preferred features. Although app-based insulin bolus calculators are available, they lack direct carbohydrate estimation and still require manual entry of meal composition or carbohydrate counts, meaning users must input this information manually rather than having it automatically recognized [5,27-31]. Separate apps can automatically count carbohydrates using photo recognition and food databases, but they do not incorporate bolus calculators [14,15,32]. This underscores participants’ perception that current CC apps lack comprehensiveness. Moreover, existing apps frequently contain unreliable values [33], which can be attributed to users contributing information to the databases or relying on values from foreign databases. Participants reiterated this concern, highlighting unreliable food databases as external sources of errors. Another barrier to CC was the associated financial burden. As such, there may also be cost-related limitations when using CC apps. The apps used by participants all offered a free version, despite the option to upgrade to a premium version.

While the benefits of CC are well established in the literature [7,8], respondents perceived a limited use of CC when it comes to explaining BG levels and glycemic trends due to the multiple dietary and nondietary factors influencing BG levels. Similarly, a Canadian survey reported similar findings and revealed that 78% of the respondents (n=180) agreed that BG levels fluctuate even with appropriate CC, complicating diabetes management [34]. The most significant barrier reported was the difficulty in accurately identifying and counting carbohydrates. In fact, several research studies documented no significant changes in CC accuracy despite receiving education [4,35]. Additionally, more than half of this survey’s respondents found determining insulin doses for high-fat meals to be a barrier to CC. Managing the delayed impact of fat on postprandial BG excursions is a well-established challenge in the literature [36-38]. Although clinical guidelines recommend meal-time insulin dose adjustments to mitigate glucose variability due to the impact of dietary fat [39-41], there is no consensus as to which insulin strategies to use. Strategies used to manage glycemic excursions following high-fat meals in T1D are numerous [42] but substantial interindividual differences exist in insulin dose requirements for fat and individualized advice based on postprandial BG monitoring for multiple hours afterward is required [43]. As such, the use of a CC app offering the ability to track meal composition, as well as other nondietary influencing factors can help people with T1D understand BG levels and allow for personalized learning opportunities. Furthermore, machine learning models that use personalized insights from tracking apps could offer new opportunities for insulin dosage recommendations that account for protein and fat intake. Although the suggested psychosocial barriers received a lower agreement rate, they are still noteworthy as they were brought up in a different light by participants in the open-ended responses. Although CC in the presence of others is not as consequential, the fear of receiving judgment from others or feeling alienated, especially when eating sugary foods, was perceived as a barrier to CC. This was also demonstrated in a cross-sectional analysis of people with T1D perceiving stigma mostly as blame and judgment, including when eating sugary foods [44]. Only 69% of participants reported an agreement with app features providing support from health care professionals. The interpretation of this finding can be informed by qualitative analysis shedding light on stigma perception related to interactions with health care providers. Participants expressed feeling judged based on their BG values, which generated a fear of “ruining a good day,” further complicating their condition management. This sentiment aligns with barriers mentioned by participants regarding the limited support from their health care team, who heavily focused on glycated hemoglobin levels rather than overall health and had limited knowledge of nutrition. They expressed the need for personalized learning opportunities that could be provided through CC apps by tracking their own trends and accessing educational modules while maintaining their privacy.

A final consideration is the potential for these features to improve CC and overall glycemic management while reducing the burden of CC. Given the challenges with CC around mealtimes, the need for technological solutions is evident. A recent meta-analysis, published in 2023, examining the effects of smartphone apps on glycemic control in youths with T1D found that apps with insulin or carbohydrate calculators were effective in reducing glycated hemoglobin (HbA_1c_) levels [45]. In addition to improving accuracy, CC apps can help individuals understand their glycemic trends and how various factors impact their BG levels. Such an app can empower users to learn about their glycemic patterns and take control of their diabetes management, particularly in contexts where patient-practitioner communication may be limited. A CC app can streamline access to relevant information for health care professionals while enabling users to track and share key details for more meaningful discussions about their care.

### Strengths and Limitations

Our results highlight the perceived barriers and preferred app features among individuals with T1D and should be interpreted with caution due to certain limitations. First, this study faced selection bias due to convenience sampling rather than random selection. Participants who completed the survey were likely more motivated, which may have resulted in an underrepresentation of certain barriers and app feature preferences. This limits the generalizability of the results, as the findings may not accurately reflect the diverse experiences of all individuals with T1D. Further, the homogeneous sample, with around 84% of the respondents identifying as Caucasian is not representative of most people with T1D and prevents the generalizability of the results. Nevertheless, 16% of this survey’s respondents self-identified as Arabs, Asians, Blacks, and Latin Americans, among other ethnicities, as compared to 26% of Canadians self-identified as belonging to visible minorities according to the 2021 census by Statistics Canada [46]. Further, participant recruitment was extended to include adolescents as well as adults. This allowed including the perceptions of young adults who are transitioning to assuming responsibility for managing their diabetes from caregivers. Second, while surveys as a research method impose limitations on participants’ responses and feedback, this was mitigated by incorporating open-ended questions. The thematic analysis provided a deeper understanding of participants’ perceptions. Third, patient-reported outcomes brought additional value to our data and shed light on patient perspectives. Further qualitative studies with in-depth interviews could provide a better understanding of the diverse experiences of people with T1D, especially considering variations in treatment plans and differences between individuals using CGMs versus self-monitoring of BG levels, as most respondents in this survey were CGM users.

### Future Directions

This study highlights the challenges faced by people with T1D and areas for improvement in diabetes management apps. Effective apps need comprehensive, automated CC features like photo recognition, easy access to frequently consumed meals, reliable nutrient values, and personalized bolus calculations. Personalization options, such as insulin administration modalities and individual factors affecting BG levels, are also essential for a user-friendly experience. An app incorporating these elements and validated food databases would be unique and novel. Ongoing end-user engagements in development and testing are essential for the high usability of the app.

## Appendix

Multimedia Appendix 1 Survey questions.

Multimedia Appendix 2 Theme description derived from the qualitative analysis.

## Abbreviations

BG: blood glucose
CC: carbohydrate counting
CGM: continuous glucose monitoring
T1D: type 1 diabetes

## Data Availability

The datasets used and analyzed during this study are available from the corresponding author on reasonable request.
