# Supplementary material for: Bridging the Gap in Carbohydrate Counting With a Mobile App: Needs Assessment Survey
Source: J Med Internet Res. 2025 Mar 28;27:e63278. doi: 10.2196/63278 (PMC11992487; doi:10.2196/63278)
Supplement: Multimedia Appendix 1 [file jmir_v27i1e63278_app1.docx]

**Appendix A: Survey questions**

# Demographic intake

1.     What is your date of birth? (Format: DD/MM/YYYY)

[TEXTUAL]

 2.     What gender do you identify with?

1.     Man

2.     Woman

3.     I prefer not to answer

4.     I don't know

**5.**     **Other: *specify***

3.     Which ethnic group do you identify with the most?

1.     White/Caucasian

2.     Black (African, Afro-american, Caribbean, etc.)

3.     Latin American

4.     Arab

5.     Asian

6.     Indigenous (First Nation)

7.     I prefer not to answer

8.     I don't know

**9.**     **Other: *specify***

4.     Were you born in Canada?

1: Yes

2: No. ***Specify***

3: I prefer not to answer

4: I don't know

 5.     If applicable, in what year did you become a landed immigrant in Canada? (Format: YYYY)

[TEXTUAL]

6.     Indicate the highest level of education you acquired.

1: Secondary 2 (grade 8) or lower

2: Secondary 3 or 4 (grade 9 or 10)

3: Highschool diploma (grade 11, 12 or 13)

4: Vocational school

5: Diploma from CEGEP or community college

6: University certificate

7: Bachelor's degree

8: Graduate studies (master's or PhD)

9: I prefer not to answer

10: I don't know

7.     If you are currently studying, indicate at what level.

1: Secondary 2 (grade 8) or lower

2: Secondary 3 or 4 (grade 9 or 10)

3: Highschool diploma (grade 11, 12 or 13)

4: Vocational school

5: Diploma from CEGEP or community college

6: University certificate

7: Bachelor's degree

8: Graduate studies (master's or PhD)

9: I am not currently studying

10: I prefer not to answer

11: I don't know

# Diabetes management intake

8.     In what year were you diagnosed with type 1 diabetes? (Format: YYYY)

[TEXTUAL]

9.     What do you use to administer yourself insulin?

1.     Insulin pump exclusively

2.     Insulin pen or syringe exclusively

3.     Sometimes with an insulin pump

4.     I do not take insulin

5.     I prefer not to answer

6.     I don't know

**7.**     **Other: *Specify***

# 10.  If you use an insulin pump, which functionalities of your insulin pump did you use in the last week? (Check all that applies)

1.     The bolus wizard or calculator (suggests a dose to administer based on blood sugar levels, amount of carbohydrates in the meal and active insulin/insulin on board)

2.     A temporary basal rate

3.     The programming of basal rates (changes in basal settings)

4.     Extension or division of insulin bolus infusion (e.g., dual wave bolus and extended bolus)

5.     Interruption or suspension of insulin infusion

6.     Set a temporary sensor glucose (SG) target (Temp Target) (e.g., sleep or sport mode)

7.     Connection of my CGM to my pump (automated insulin pump)

8.     I did not use any of these functions

9.     I prefer not to answer

10.  I don't know

**11.**  **Other: *Specify***

11.  How many rapid-acting insulin injections (or insulin pump deliveries) do you take in a typical day?

1.     1-2 insulin injection per day

2.     3-4 insulin injections per day

3.     5-6 insulin injections per day

4.     7 insulin injections per day or more

5.     I prefer not to answer

6.     I don't know

12.  In the last 12 months, which continuous glucose monitoring (CGM) device did you use?

1.     The Dexcom G6

2.     The Freestyle Libre system from Abbott

3.     The Freestyle Libre 2 system from Abbott

4.     The Medtronic's Guardian Sensor 3

5.     The Medtronic’s Guardian Connect

6.     I don’t use a continuous glucose monitoring (CGM) device

7.     I prefer not to answer

8.     I don't know

9.     **Other: *Specify***

 13.  In the past year, what is the frequency to which you saw the following healthcare professional (s) to consult for your diabetes? (Enter digits only where applicable)

1.     Medical specialist (Endocrinologist, Pediatrician, Internist): ## times /year

2.     Family doctor (General practitioner): ## times /year

3.     Dietitian/Nutritionist: ## times /year

4.     Nurse: ## times /year

5.     I prefer not to answer

6.     I don't know

**7.**     **Other: specify ## times /year**

# Perceived usefulness and barriers in carbohydrate counting

14.  Is your rapid-acting insulin dose given at mealtime (i.e., bolus) determined by the quantity of carbohydrates in your meals and/or snacks (insulin-to-carb ratio): e.g., 1 unit of insulin per 10 grams of carbohydrates consumed)?

1.     Yes

2.     No

3.     I don't know

4.     I prefer not to answer

15.  How often do you use carbohydrate counting to match insulin doses with carbohydrate content? (Select the answer closest to your reality)

1.     At every meal and snack

2.     For some meals and snacks during the day

3.     Once a day

4.     For some meals during the week

5.     Once a week

6.     Less than once a week

7.     Never

 16.  To what extent do you agree or disagree with the following statements considering the following scale: (1) Strongly disagree; (2) Disagree; (3) Neither agree nor disagree; (4) Agree; (5) Strongly agree.

**One of the barriers to carbohydrate counting is:**

1. Identifying the amount of carbohydrates when unsure of how much I will eat (buffet dinner, unsure about appetite)
2. Identifying the amount of carbohydrates in foods without labels
3. Identifying the amount of carbohydrates in foods in restaurants and when eating out
4. Determining insulin doses for meals with protein-rich foods
5. Determining insulin doses for meals with fat-rich foods
6. Carbohydrate counting in the presence of others (social stigma)
7. The burden of treatment (i.e., To purposely avoid counting all carbohydrates to simplify the calculation)
8. Continuously monitoring your blood glucose levels to adjust insulin-to-carbohydrate ratio

17.  Specify, if any, other barriers to carbohydrate counting?

[TEXTUAL]

18.  To what extent do you agree or disagree with the following: It is difficult to control blood glucose levels around mealtime even with carbohydrate counting.

1.     Strongly Agree

2.     Agree

3.     Neither Agree nor Disagree

4.     Disagree

5.     Strongly Disagree

19.  How often do you use a phone or tablet app or websites to help you with carbohydrate counting? (Select the answer closest to your reality)

1.     At every meal

2.     Most of the days

3.     Some days

4.     Rarely

5.     Never

**If you are currently using app (s) for carbohydrate counting, answer the following 3 questions.**

20.  Enter the name of the app (s) that you most often use to help you with carbohydrate counting.

[TEXTUAL]

21.  What do you like the most in the app (s) that you are using for carbohydrate counting?

[TEXTUAL]

22.  To what extent do you agree or disagree with the following: The app (s) you are currently using meet (s) your needs in carbohydrate counting.

1.     Strongly Agree

2.     Agree

3.     Neither Agree nor Disagree

4.     Disagree

5.     Strongly Disagree

**If an application designed to help you with carbohydrate counting was developed,**

 23.  To what extent do you agree or disagree with the following:

**Features that would be beneficial to include in a carbohydrate counting app are:**

1. Meal logs
2. Quantification of meal composition (total amount of meal portion, amount of protein, fat, carbohydrate)
3. Automated calculation of bolus doses using a CGM glucose value (based on your insulin to carb ratio, and number of carbohydrates entered)
4. Personalization of the app (food log parameters, carbohydrate counting and insulin dose suggestions)
5. Viewing the food nutrition label
6. Gamification features (earning points/trophies, tracking progress towards an objective...)
7. Support from healthcare professionals (i.e., Q&A)
8. Ability to build a community with other users in the app (i.e., texting, interacting with others on the app)

24.  Specify, if any, other features that would be beneficial to include in a carbohydrate counting app:

[TEXTUAL]

 25.  To what extent do you agree or disagree with the following statements:

**Factors that would increase my trust in an app designed for carbohydrate counting and insulin dose calculation are:**

1. Validated results from clinical trials.
2. Secure data storage.
3. Information on safety mechanisms included in the app to avoid accidental overdosages/hypoglycemia (i.e., where are the suggestions coming from)
4. Having access to the mathematical formulas or algorithms used in generating the insulin dose suggestions (how was the suggestion generated based on preferred setting i.e., insulin-to-carb ratio, formula).
5. Qualifications of the developers of the app.
6. Health Canada’s approval.
7. Support from my healthcare team in using the carbohydrate counting app (i.e., my healthcare professional endorses the app, helps me navigate the app).

26.  Specify, if any, other factors that would increase your trust in an app designed for carbohydrate counting and insulin dose calculations:

[TEXTUAL]

27.  Do you believe that using an app to help you with carbohydrate counting can be a source of motivation – ease of use and ease the burden for your diabetes management?

1.     Strongly Agree

2.     Agree

3.     Neither Agree nor Disagree

4.     Disagree

5.     Strongly Disagree

# Perceived usefulness and barriers in food journaling

 28.  To what extent do you agree or disagree with the following: it is difficult to keep a food log.

Strongly Agree

Agree

Neither Agree nor Disagree

Disagree

Strongly Disagree

 29.  To what extent do you agree or disagree with the following statements:

**What makes keeping a food log difficult is:**

1. Forgetting to log meals
2. It is time-consuming to log meals
3. It is difficult to determine what I ate (ingredients or food composition)
4. It is difficult to quantify how much I ate
5. Food database or looking up food on the Internet is not always reliable
6. Recording food not found in the database (Homemade, bought at a locally owned restaurant…)
7. Continuously updating your food logs throughout the day

30.  Specify, if any, other factor that make keeping a food log difficult:

[TEXTUAL]

**If an application designed to help you with food journaling was developed,**

31.  To what extent do you agree or disagree with the following statements

**Features that would facilitate food journaling are:**

1. Receiving optional reminders for forgotten logs
2. Photo recognition (Ability to take a picture of the meal for food entry)
3. The option to save meals in a *Favorite* section for food entry
4. The option to view recent meal items in a *History* section.
5. The option to combine food items into recipes and save them in the app (e.g., peanut butter toast vs toasted bread + peanut butter).
6. The ability to view blood glucose levels with related meals and insulin doses/correction factors.
7. The ability to adjust meal data and add notes (type, amount, specification about food, mealtime behaviours…).
8. The ability to log other information (Alcohol intake, physical activity, insulin on board, health events, medications, meal fat and protein content…).

32.  Specify, if any, other feature (s) that would facilitate food journaling:

[TEXTUAL]

# Perceived usefulness and barriers in sharing food and blood glucose trends with healthcare team

 To what extent do you agree or disagree with the following statements:

 33.  It is important for you to share your food log and blood glucose information with your healthcare professional.

1.     Strongly Agree

2.     Agree

3.     Neither Agree nor Disagree

4.     Disagree

5.     Strongly Disagree

It is difficult for you to share your food log and blood glucose information with your healthcare professional.

1.     Strongly Agree

2.     Agree

3.     Neither Agree nor Disagree

4.     Disagree

5.     Strongly Disagree

34.  What aspects can make communicating your diabetes information (food logs and blood glucose levels) with your healthcare professional difficult? (Check all that applies)

1.     Burdensome to record food and blood glucose logs

2.     Forgetting to bring/upload blood glucose logs and food logs to medical appointment

3.     Difficult to recall meals for specific blood glucose levels.

4.     Difficult to recall specific factors (i.e., food composition, infections, physical activity) for blood glucose levels.

5.     Difficult to keep and store all the food and blood glucose logs until medical appointment with healthcare professional

6.     None

7.     I prefer not to answer

8.     I don’t know

9.     Other: specify

 35.  Do you use technology (apps, platforms, websites) to help you share your food and blood glucose logs with your healthcare professional?

1.     At every appointment

2.     Most of the appointments

3.     Some appointments

4.     Rarely

5.     Never

36.  If you are using a phone or tablet app to help you share your diabetes management data with your healthcare professional, enter the name of the app (s) below

[TEXTUAL]

**If an application designed to help you communicate your diabetes management data with your healthcare provider was developed,**

37.  What information would you like to be able to share with your healthcare provider? (Check all that applies)

1.     Food journal

2.     Blood glucose levels

3.     Other logged events (Physical activity, stress levels, sick days, alcohol intake…)

4.     None

5.     I prefer not to answer

6.     I don’t know

7.     Other: specify

38.  How do/*would* you use technology to share your diabetes information with your healthcare professional? (Check all that applies)

1.     Generating reports to download

2.     Generating reports to send them by email directly through the app

3.     Allowing total or partial access directly through a healthcare professional portal (i.e., using a code or link)

4.     I prefer not to answer

5.     I don’t know

6.     Other: Specify

 39.  Please leave any other comments you may have.
