# Supplementary material for: Bridging the Gap in Carbohydrate Counting With a Mobile App: Needs Assessment Survey
Source: J Med Internet Res. 2025 Mar 28;27:e63278. doi: 10.2196/63278 (PMC11992487; doi:10.2196/63278)
Supplement: Multimedia Appendix 2 [file jmir_v27i1e63278_app2.docx]

Appendix B: Theme description derived from the qualitative analysis.

| **Codes BARRIERS** |
| --- |
| **Non-dietary factors affecting glycemia** (physical activity, stress, sick days, medication, jetlag, weather, alcohol, sleep, menopause, menstrual cycle, honeymoon period, gastroparesis, hypo/hyper before meals) |
| **Meal composition influences on BG** (Glycemic index, fat-rich foods, protein-rich foods) |
| **Quantifying portions** (homemade food, mixed meals, unknown ingredients, restaurants/workplace/eating out, unclear serving size on nutrition labels e.g. “1/10 of the package”) |
| **Mental burden** (time consuming, remembering meal/insulin given, logging multiple small snacks, logging corrections outside of meals, fatigue, financial burden, fear of consequences, not being able to eat right away, having to carry a physical journal/scale not realistic, lack of motivation) |
| **Sources of errors** (calculation mistakes, unreliable nutrition labels, unpredictability in results, distractions, discarding packaging, long meals, not finishing meals, lack of reliable sources of nutrient values) |
| **Stigma** (receiving/fear of receiving judgment from others when eating sugar foods/self/HCP, own preoccupation about others, fear of ruining a "good day", feeling alienated) |
| **Eating disorders or fear of developing disordered eating** (hyper-fixation on food, forced to eat, forced to stop eating, forced to have a meal schedule vs improvised meals) |
| **Limited use of CC apps** (unreliable, no comprehensive app, not satisfied yet with current apps, bugs, partial use of CC app only when needed vs regularly, limited impact of CC app on BG and reducing burden, limited access, requires more time spent on screens, takes too long/difficult to use) |
| **Limited support from HCP** (difficult to share data, limited time during appointments, focus on high glycated hemoglobin only vs overall health, limited knowledge in Nutrition, lack of understanding of patient perspective, fear of invasion of privacy) |
| **Limited perceived benefit from CC** (CC too burdensome to care about CC, no utility of CC on explaining BG trends, no impact of CC on improving BG) |
| **Other strategies** (Avoiding carbs, using CGM to correct, using smart scales to generate food labels, using pumps for bolus calculation, time of injection before OR after, eating the same thing, "guesstimating"/trial and error) |
| **Codes APP-FEATURES** |
| **Simple interface and rapid data entry** (bar code, large database for meal entry, local food, restaurant food items/brands, option for manual entry/edit, adding custom recipes, favorites/history sections, copy and paste meal entries) |
| **AI-Photo recognition** |
| **Reliable nutrient value** (net carbs, kcal, fibers, macro, micronutrients, GI, source of info included, breakdown of estimations) |
| **Promote a healthier relationship with food** (Concealing calorie information) |
| **Portion estimations** (visual cues for portion size, taking a picture of food label to upload in the app, different options: food weight, units, cups, mg, portions, photos for comparison, small/medium/large vs exact quantity) |
| **Tracking factors affecting glycemia** (meals, stress, physical activity, sick days, menstrual cycle, sleep, time of injections, site of injection, other daily events or notes, graphic trends, week/month-overview, considers other medical factors) |
| **Personalized learning opportunities** (suggestions based on BG/food preferences/stage of life, adding objectives non-related to diabetes management, gamification i.e., earning points/winning prizes, setting reminders, education content on fat-rich foods/protein-rich foods/new technology, learning from personal trends) |
| **Peer support** (discussion groups) |
| **Reliable and personalized bolus calculations** (automatic calculation to be validated by users, manual adjustments based on own experience, correction units suggested, considers correction factors and I:C ratio re time of day, considers active insulin, considers PA/energy expenditure, considers high fat and high protein meals, recommendations to adjust I:C ratio/timing and dosing of insulin, option to add a sensitivity-to-insulin scale to account for non-dietary factors based on experience) |
| **Integration with other devices/software** (Siri, CGM, glucometers, pumps, pens, HCP portals, compatibility with different phones, ability to retrieve CGM and pump info uploads from the app, smart scales) |
| **Evidence-based CC app by and for people with T1D** (designed by people with T1D, for people with T1D, endorsed by HCP and diabetes organization, partnership with trusted establishments, clinical trials, users feedback, consistency in results obtained from app, improved BG outcomes, up to date with no unnecessary delays to obtain Health Canada approval as an example, secure and private, large number of users) |
